# Measuring the Influence of Noise Reduction on Listening Effort in Hearing-Impaired Listeners Using Response Times to an Arithmetic Task in Noise

**DOI:** 10.1177/23312165211014437

**Published:** 2021-05-24

**Authors:** Ilja Reinten, Inge De Ronde-Brons, Rolph Houben, Wouter Dreschler

**Affiliations:** 1 Clinical & Experimental Audiology, Amsterdam University Medical Centres location AMC, Amsterdam, the Netherlands; 2 Pento Audiological Centre, Utrecht, the Netherlands; 3 Pento Audiological Centre, Amersfoort, the Netherlands

**Keywords:** hearing loss, hearing aids, perception, noise, cognitive resources, FUEL model for listening effort

## Abstract

Single microphone noise reduction (NR) in hearing aids can provide a subjective benefit even when there is no objective improvement in speech intelligibility. A possible explanation lies in a reduction of listening effort. Previously, we showed that response times (a proxy for listening effort) to an auditory-only dual-task were reduced by NR in normal-hearing (NH) listeners. In this study, we investigate if the results from NH listeners extend to the hearing-impaired (HI), the target group for hearing aids. In addition, we assess the relevance of the outcome measure for studying and understanding listening effort. Twelve HI subjects were asked to sum two digits of a digit triplet in noise. We measured response times to this task, as well as subjective listening effort and speech intelligibility. Stimuli were presented at three signal-to-noise ratios (SNR; –5, 0, +5 dB) and in quiet. Stimuli were processed with ideal or nonideal NR, or unprocessed. The effect of NR on response times in HI listeners was significant only in conditions where speech intelligibility was also affected (–5 dB SNR). This is in contrast to the previous results with NH listeners. There was a significant effect of SNR on response times for HI listeners. The response time measure was reasonably correlated (*R*_142_ = 0.54) to subjective listening effort and showed a sufficient test–retest reliability. This study thus presents an objective, valid, and reliable measure for evaluating an aspect of listening effort of HI listeners.

It is well known that single microphone noise reduction (NR) in hearing aids (HAs) can lead to a subjective benefit, in terms of listener preference, even when there is no objective improvement in speech intelligibility. Brons et al. (2014a) evaluated several NR algorithms in commercially available HAs by performing speech intelligibility tests and quality ratings in 12 hearing-impaired (HI) subjects. The HAs that performed best in terms of listener preference had the worst speech intelligibility scores. Chung et al. (2009) evaluated a modulation-based digital NR algorithm in terms of sound quality and speech intelligibility with several background noise types. Their study with 16 normal-hearing (NH) listeners showed that listeners preferred NR even when speech intelligibility was the same. This suggests that in addition to speech intelligibility, there are other factors that determine listener preference for NR.

A plausible explanation is that NR can establish a reduction in listening effort. Listening effort can be described as a complex interaction of the cognitive resources that are required for adequate speech understanding combined with the motivation to succeed in a listening task in a particular surrounding (Pichora-Fuller et al., 2016). Understanding speech in a noisy environment requires more cognitive effort than understanding the same speech in a quiet environment. This requirement for additional effort can cause fatigue and annoyance in the listener. As a consequence, it can eventually lead to a loss of attention. Conversely, a listener who is initially inattentive or unmotivated can perceive a listening situation as more effortful even though the environment is optimal. Furthermore, for listeners with hearing loss, listening effort is generally larger than for NH listeners (Ohlenforst et al., 2017). The value of NR in HAs for the end-users does not stem from improvement in speech intelligibility (which it does not improve) but might lie in a reduction in listening effort. A reliable and valid method of measuring the effect of NR on listening effort is lacking.

There are several studies in which measures of listening effort have been investigated. Physiological measures of listening effort include pupil dilation response (Ohlenforst et al., 2018), heart rate variability (Mackersie & Calderon-Moultrie, 2016), and electroencephalogram recordings (Obleser et al., 2012). Other commonly used methods to study listening effort are self-report measures (Brons et al., 2013; Rudner et al., 2012) or behavioral measures (Ng et al., 2015; Rönnberg et al., 2014). An overview of the most used measures can be found in McGarrigle et al. (2014). Although these methods were originally developed to each serve as a proxy for listening effort, it is becoming clear that listening effort cannot be captured by a single measure. Alhanbali et al. (2019) compared several measures and found that they were poorly correlated to each other, although the reliability within each measure was high. This finding supports the emerging idea that listening effort is a broader concept that captures several aspects of effortful listening.

In an attempt to describe the multidimensionality of listening effort, Pichora-Fuller et al. (2016) proposed a Framework for Understanding Effortful Listening (FUEL). This framework describes the effort required to successfully complete a listening task as a combination of two aspects: task-demand and motivation. The model categorizes outcome measures for listening effort into cognitive-behavioral measures, brain activity measures, autonomic nervous system activity measures, or self-reported sound quality ratings. The authors suggest that cognitive-behavioral tests measures are related to the task-demand part of listening effort and that physiological measures are related to the motivational part of listening effort (Pichora-Fuller et al., 2016; Rudner, 2016). However, certain measures might tap into both aspects of listening effort. In the current study, we focus on a cognitive-behavioral approach to measuring the effect of NR on listening effort.

A promising cognitive-behavioral approach to measuring listening effort is a dual-task paradigm. Dual-task paradigms assume that there is a limit to the available cognitive resources (Kahneman, 1973). To measure listening effort, a primary listening task is combined with a simultaneous secondary task that requires cognitive processing. When the primary task is more demanding, the cognitive spare capacity of the listener is reduced. This reduction leads to decreased performance on the secondary task (Gosselin & Gagné, 2010; Hicks & Tharpe, 2002). The reduction in performance level on the secondary task is an indication of the listening effort that is required for the primary task.

For the measurement of listening effort, an auditory primary task can be combined with a nonauditory secondary task. An example of a nonauditory secondary task is a reaction to a visual cue (Desjardins & Doherty, 2014; Sarampalis et al., 2009). Sarampalis et al. (2009) tested response times to a visual cue (secondary task) while listening to speech in noise at different signal-to-noise ratios (SNR; primary task). They found that at –6 dB SNR, NH listeners responded faster when the primary task stimuli were processed with an NR algorithm based on a minimum mean square estimator (MMSE; Ephraim & Malah, 1984). Desjardins and Doherty (2014) tested performance in a secondary visual tracking task in moderate and difficult listening situations. The authors found that in difficult listening situations, HI listeners performed better on the secondary task when NR from a commercially available HA was applied to the speech in the primary task. However, in listening conditions where speech was fully intelligible, both studies found no effect of NR on performance.

The secondary task can be designed to make use of the outcome of the primary listening task. Strictly speaking, this does not qualify as a dual-task paradigm as the two tasks are not performed simultaneously. Nonetheless, the secondary task does require additional cognitive resources that could be reduced when the listening conditions of the primary task are more difficult. An advantage of such an auditory-only setup is that it requires less equipment and is therefore better suited for clinical applications. In addition, test results are not influenced by a possible nonauditory sensory impairment (e.g., a visual impairment) in the listener that might not be immediately evident in a clinical setting. Houben et al. (2013) designed an auditory-only dual-task with digit triplet stimuli in noise with different SNRs. The primary task was to identify the digits, and the secondary task was to add the first and third digit. The authors showed that for NH listeners, response times to this simple secondary arithmetic task reduced with increasing SNR on the primary task. Moreover, this reduction in response times in more favorable listening conditions was also present for conditions where speech intelligibility was at its maximum (100% correct). Therefore, this particular measure seems applicable for quantifying effortful listening in SNRs where speech intelligibility fails to demonstrate a potential benefit.

In a follow-up study, van den Tillaart-Haverkate et al. (2017) used the previously described method for evaluating the effect of different forms of NR processing on response times. The authors hypothesized that NR shortens response times by reducing the cognitive load in listening situations with maximum speech intelligibility. Indeed, in a group of 12 NH listeners, they found a significant reduction in response time due to the application of NR. That study yielded promising results showing that it is possible to assess listening effort by means of a dual-task paradigm based on auditory-only stimuli. However, the effect of NR on listening effort must be investigated in HI listeners, as the intended users of HAs. HI listeners can have significantly different opinions regarding the sound quality of HA features compared with NH listeners (Huber et al., 2018) and that might be related to different levels of listening effort in HI listeners.

In the present study, we investigated whether NR reduces listening effort in HI listeners. We also investigated the relevance of response times to an auditory-only dual-task as an outcome measure for studying and understanding listening effort. Twelve HI listeners participated in an auditory-only dual-task listening test to (a) determine the effect of NR on response times and (b) to study two important aspects of an outcome measure: the validity and reliability. To test validity, we included a self-report listening effort measure. To test the reliability of the measure, we repeated all measurements after 2 to 4 weeks. We place the results in the context of the FUEL model for listening effort.

## Methods

This study was approved by the Medical Ethics Committee of the Amsterdam UMC (former AMC) in 2013 (MEC2013_082). All participants signed an informed consent before starting with the experiment.

### Participants

**Figure 1.**
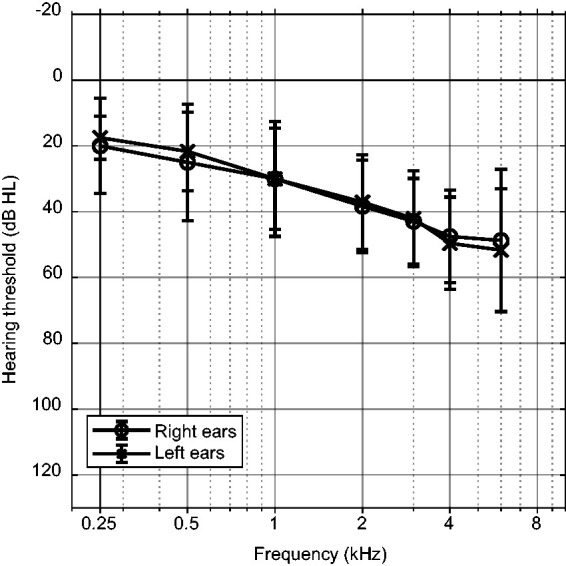
Group-Averaged Pure Tone Thresholds of the HI Subjects With Interindividual Standard Deviations. HL = hearing level.

Twelve HI listeners participated in this experiment. The HI listeners had a mean age of 60 ± 5.3 years and had a symmetric mild to moderate sensorineural hearing losses. The group-averaged pure tone thresholds of the included participants are shown in Figure 1.

The following were auditory inclusion criteria: a pure tone threshold between 30 and 70 dB hearing level at 4 kHz with an air–bone gap smaller than 15 dB in the frequency range from 250 to 4000 Hz. All participants were over 18 years old and were native Dutch speakers. Six participants used HAs in daily life, three of whom had been using them for more than 5 years. The participants were recruited in the Audiological Centre of the Amsterdam UMC, location AMC.

### Stimuli and Processing

We used 60 spoken digit triplets of the Dutch digit triplet test (Smits et al., 2013). The digit triplets, spoken by a male speaker, contain digits from zero to nine in unique combinations of three digits. We used only those triplets that allowed participants to carry out the arithmetic task (explained later) with a single key-press. The digit triplets were combined with speech-shaped stationary noise to create four different SNRs: –5, 0 + 5, and +∞ (speech in quiet) dB. For speech in quiet, the average level of the speech was 65 dB (A). For the speech in noise conditions, the noise level was fixed at 65 dB(A) and the speech level was varied to create the different SNRs. At each SNR, we processed the digit triplets to create three conditions per SNR: one unprocessed (Unpr) condition and two processed conditions with two types of NR algorithms. The two NR algorithms applied in the experiment were the ideal binary mask (IBM) and an MMSE. The most prominent difference between these two algorithms is that the IBM uses a priori knowledge of the noise and speech material as separate signals and is known to be able to improve speech intelligibility (Wang et al., 2009). Because the IBM requires a priori knowledge of the noise and speech signals, it is not suitable for HA implementation. It does, however, give insight into the maximum achievable benefit offered by NR. The MMSE on the other hand estimates the SNR from the mixed signal and is therefore more comparable to NR algorithms that are implemented in HAs. Implementation of the algorithms was done in MATLAB and is described in detail in van den Tillaart-Haverkate et al. (2017), which also includes a detailed description of the stimuli and equipment used. The three processing conditions at four SNRs add up to a total of 12 conditions that were used in the experiment.

### Test Procedure

Participants completed the test battery during a single session. The test session was repeated 2 weeks later to obtain test–retest reliability estimates. The test procedure was identical during both visits, with the exception of pure tone audiometry, which was only performed at the first visit. The primary outcome measure of this experiment was response time to an arithmetic task. Secondary outcome measures were speech intelligibility and a perceived listening effort rating. All stimuli were presented diotically through headphones. The presentation of the stimuli differed from the study by van den Tillaart-Haverkate et al. (2017) in that additional amplification was applied after NR processing to compensate for the hearing loss. In this study, the amplification of the stimuli was calculated separately for each participant using linear amplification according to the NAL-RP rule (Byrne et al., 2001).

#### Arithmetic Task

For the arithmetic task, we instructed the participants to add the first and third digit of each digit triplet and answer as quickly as possible on a numerical keypad. Absolute response times were defined as the time between the end of playing the last digit and the subsequent response key-press. We used the same 60 digit triplets in noise for each of the 12 conditions, split into 2 sets of 30 triplets. Each participant started with 30 triplets for all 12 conditions, followed by a short break. They then finished with the second set of 30 triplets in all 12 conditions. The sets were balanced across conditions, and the order of conditions was balanced across participants using a Latin square design (Wagenaar, 1969). The arithmetic task was preceded by a practice session of 20 digit triplets that contained all conditions to familiarize the participants with the test and to reduce possible training effects.

#### Speech Intelligibility and Perceived Listening Effort Rating

Speech intelligibility and the perceived listening effort rating were measured for each condition in a combined listening test. The order of the 12 conditions was balanced across participants using a Latin square design. Per condition, the participant was first asked to correctly repeat 20 triplets (one triplet at a time), without any time constraint. They subsequently rated their perceived listening effort for the 20 triplets that they had just identified. Participants were asked to answer the following question: “How much effort did it take to understand the last 20 triplets?” Listening effort rating was scored on a 9-point scale ranging from *no effort* (1) to *extremely high effort* (9), based on an International Telecommunication Union (1996) recommendation.

### Data Analysis

As was done in the analyses by Houben et al. (2013) and van den Tillaart-Haverkate et al. (2017), we used linear mixed effect models to analyze the relative response times, speech intelligibility, and perceived listening effort. This allows us to test the effect of NR, SNR, and their interaction on the outcome measures with multiple random effects. In addition, linear mixed effect models can handle missing data.

For the arithmetic task, only correct responses were included in the analysis. Response time data are known to have unwanted outliers due to a loss of attention or unresponsiveness of the participant. To remove such unrealistically long responses, the highest 1.25% of all response times were considered missing data. The value of 1.25% is in line with the analyses of Houben et al. (2013) and van den Tillaart-Haverkate et al. (2017). The cutoff resulted in a removal of all responses times longer than 3.3 s. As we are interested in the effects of SNR and NR processing on response times rather than the between-participant differences in absolute response times, we used relative response times for data analysis. Relative response times were calculated by subtracting the average response time at +∞ dB SNR (speech in quiet) from each individual response time.

There were 12 conditions in total, including three conditions in quiet. Ideally, NR should be transparent for signals that do not contain noise. Acoustical analysis of the quiet conditions showed that there were no relevant differences between the three processing conditions (Unpr, MMSE, and IBM) using triplets in quiet. For the acoustical analysis, all triplets in quiet were combined into three long signals, one for each processing condition. These signals were subtracted from each other, after correcting for processing delay. Root-mean-square values of the differences were about 60 dB lower than the original root-mean-square of these signals. In addition, 5 NH listened to the quiet conditions and confirmed that the three conditions in quiet were indistinguishable. Because the quiet conditions can be considered perceptually equivalent, we used the average response times of all processing conditions in quiet, per triplet, per participant, and per visit, as reference values for the relative response times.

In the speech intelligibility test, we adhered to the appropriate scoring for the digit triplet test: Responses were considered correct only when all three digits were identified correctly (Smits et al., 2004). We calculated the percentage correct responses per condition. For statistical analysis, these percentages were transformed using the rationalized arcsine transform so as to satisfy the homoscedasticity criterion. The rationalized arcsine transform is a common transformation that is used prior to statistical analysis to mitigate skewing of the distribution due to saturation effects around 0 and 100% (Studebaker, 1985).

## Results

### Arithmetic Task

**Figure 2.**
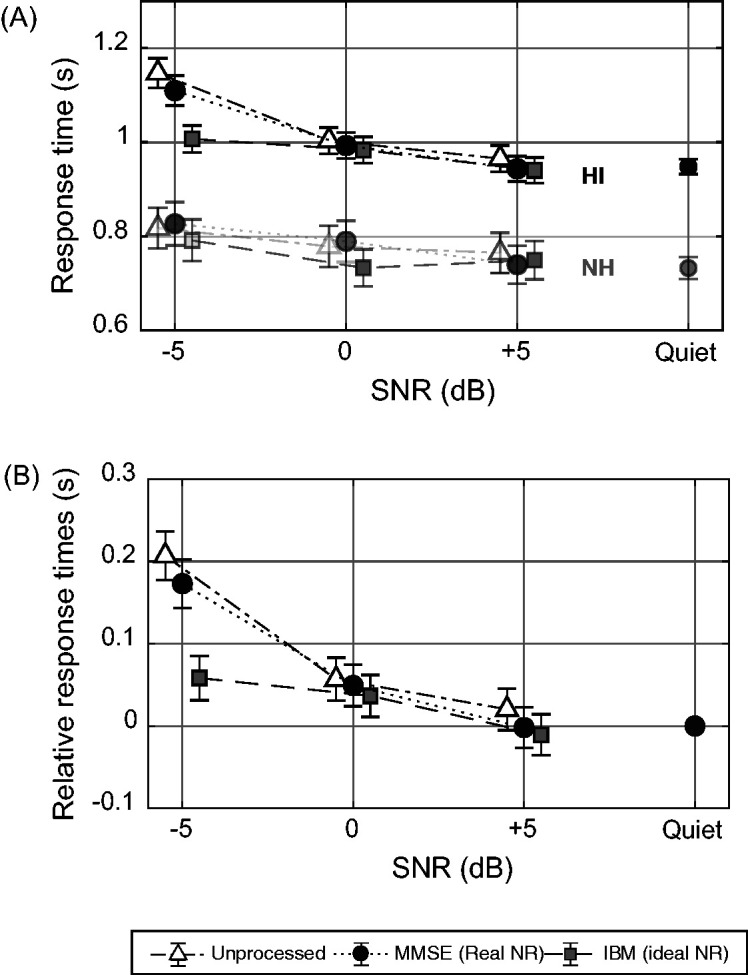
Response Times Versus SNR. (A) Absolute group-averaged response times of HI listeners. As a reference, the data of NH listeners (van den Tillaart-Haverkate et al., 2017) are plotted in the same figure. (B) Relative group-averaged response times of HI listeners. The error bars show 95% confidence intervals. NH = normal-hearing; HI = hearing-impaired; SNR = signal-to-noise ratio; MMSE = minimum mean square estimator; NR = noise reduction; IBM = ideal binary mask.

Figure 2A shows the group-averaged absolute response times for the arithmetic task for all conditions for HI listeners as well as the previously published data from NH listeners in van den Tillaart-Haverkate et al. (2017). Figure 2B shows the mean relative response times for all conditions for HI listeners. Error bars show 95% confidence intervals.

We used a linear mixed effect model that included visit, processing condition, SNR, and the interaction between processing condition and SNR as fixed effects and participant as random effects. In contrast to the analysis of Houben et al. (2013) and van den Tillaart-Haverkate et al. (2017), we did not include triplets as a random effect. Because the relative response times were calculated for each triplet separately, the effect of triplet is already included in the variation of the relative response times.

We found a significant effect of visit, *F*(1, 16504)=4.89, *p* = .027, with an effect size of *d* = 0.016. There were also significant effects of processing condition, *F*(2, 16504)=18.3, *p* < .0001; SNR, *F*(3, 16504)=91.6, *p* < .0001; and the interaction between processing condition and SNR, *F*(6, 16504) = 8.33, *p* < .0001. The between-participant variability had a standard deviation of 0.035 which explained 7.78% of the total residual variance in the model. Given the significant interaction between processing condition and SNR, we performed a post hoc analysis with Bonferroni corrections (α = .05/27). These post hoc pairwise comparisons showed that the response times at –5 dB SNR were significantly shorter for the IBM condition than for the Unpr (*p* < .0001) and MMSE (*p* < .0001) conditions. Within the Unpr and MMSE conditions, response times at –5 dB SNR were significantly longer than at all other SNRs and in quiet (*p* < .0001). In addition, within the Unpr condition, response times at 0 dB SNR were significantly longer than in quiet (*p* = .011). Within the IBM condition, response times at –5 dB SNR were significantly longer than at +5 dB SNR (*p* = .0001) and in quiet (*p* = .015).

**Figure 3.**
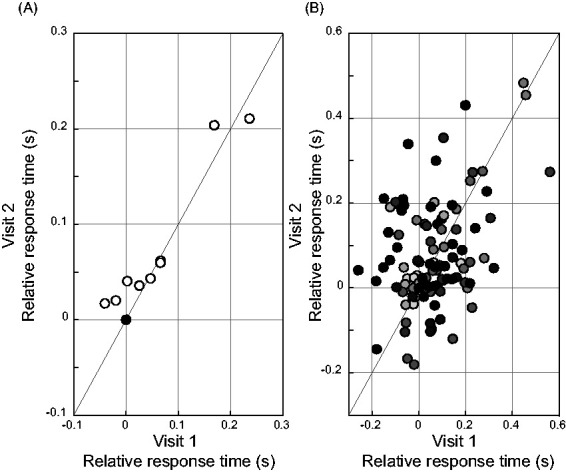
Relative Response Times Scatterplots. (A) Scatterplot of relative group-averaged response times per visit. Each marker shows the average results of one condition. The black marker consists of the three conditions in quiet, which are equal by definition in the relative response times. The *x *= *y* line is plotted in gray. (B) Scatterplot of mean relative response times per participant and per visit. Each marker shows the average result for one participant in one condition. Different participants are distinguishable by different gray tones. The *x* = *y* line is plotted in gray.

A Pearson’s product moment correlation measurement of the group-averaged relative response times per condition in the first and second visit resulted in a correlation coefficient of *R*_10_=0.95, *p* < .0001. Figure 3A shows a scatterplot of the group-averaged relative response times per condition of Visit 1 versus Visit 2. Because the three conditions in quiet were equalized in the relative data, three of the data points in Figure 3A overlap. The black marker represents the three equal data points. If these data points would be considered as one, the Pearson’s product moment correlation measurement would result in a correlation coefficient of *R*_8_=0.96, *p* < .0001. A Pearson’s product moment correlation measurement of the mean relative response times of each participant per condition in the first and second visit resulted in a correlation coefficient of *R*_142_=0.42, *p* < .001. Figure 3B shows a scatterplot of the mean relative response times for each participant per condition of Visit 1 versus Visit 2. Each marker represents the result of one condition per participant. Different participants are marked with a different gray tone.

On average, 96% of all sums were answered correctly. The most incorrect responses were given at –5 dB SNR for the Unpr stimuli and the MMSE stimuli. There was a significant difference in accuracy between visits, *F*(1)=8.96, *p* = .003; 95% of the sums in Visit 1 were answered correctly compared with 97% of the sums in Visit 2. The pattern of correct responses for the arithmetic task is similar to the results of the speech intelligibility task. Therefore, the correct responses to the arithmetic task do not provide additional information over and above the speech intelligibility measure. All incorrect responses were considered missing data. However, it should be noted that had the incorrect responses been included, none of the results reported as being significant would have changed.

### Speech Intelligibility and Listening Effort Rating Tests

**Figure 4.**
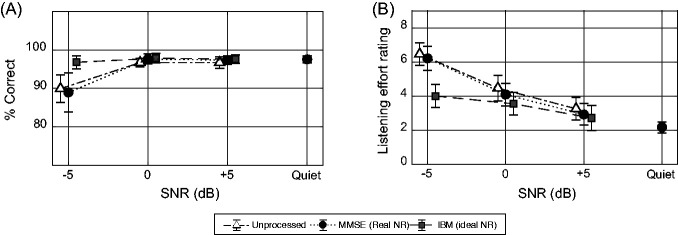
Speech Intelligibility and Perceived Listening Effort Rating Results. (A) Group-averaged speech intelligibility in terms of % correct responses for all SNRs and processing conditions; the error bars show 95% confidence intervals. (B) Group-averaged perceived listening effort rating for all SNRs and processing conditions; the error bars show 95% confidence intervals. SNR = signal-to-noise ratio; MMSE = minimum mean square estimator; NR = noise reduction; IBM = ideal binary mask.

Figure 4A shows the group average results of the speech intelligibility test in terms of the % correct identification scores of triplets. We analyzed the speech intelligibility with a mixed model analysis of variance on the rationalized arcsine unit-transformed intelligibility scores, with subject and triplet as random effects. Processing condition, SNR, and the interaction between processing condition and SNR were considered fixed effects. We found significant effects of processing condition, *F*(2, 121)=12.33, *p* < .0001; SNR, *F*(3, 121)=35.82, *p* < .0001; and the interaction between processing condition and SNR, *F*(6, 121)=5.08, *p* = .001. The between-participant variability had a standard deviation of 1.18 which explained 52.2% of the total residual variance in the model. Post hoc pairwise comparisons after Bonferroni corrections (with α = .05/27) of the conditions revealed that at –5 dB SNR the IBM condition was significantly more intelligible than the Unpr condition (*p* = .001). Within the Unpr and MMSE conditions, intelligibility at –5 dB SNR was significantly worse than at all other SNRs (*p* < .001). Within the IBM condition, intelligibility did not differ significantly between the SNRs.

Figure 4B shows the group average result for the subjective rating of perceived listening effort. We used a linear mixed effect model for the perceived listening effort rating data that included visit, processing condition, SNR, and the interaction between processing condition and SNR as fixed effects and participant as random effects. We found a significant effect of processing condition, *F*(2, 265)=19.22, *p* < .0001; SNR, *F*(3, 265)=139.24, *p* < .0001; and the interaction between processing condition and SNR, *F*(6, 265)=8.24, *p* < .0001. Post hoc analysis after Bonferroni corrections (with α = .05/27) revealed that at –5 dB SNR, the IBM condition was rated significantly less effortful than the Unpr (*p* < .0001) and MMSE (*p* < .0001) conditions. Within the Unpr and MMSE conditions, the perceived listening effort rating significantly increased with decreasing SNR, except between 5 dB SNR and quiet for the MMSE condition. Within the IBM condition, perceived listening effort rating at –5 dB SNR was significantly higher than at 5 dB SNR (*p* = .002) and quiet (*p* < .0001), and the perceived listening effort rating at 0 dB SNR was significantly higher than in quiet (*p* = .002).

**Figure 5.**
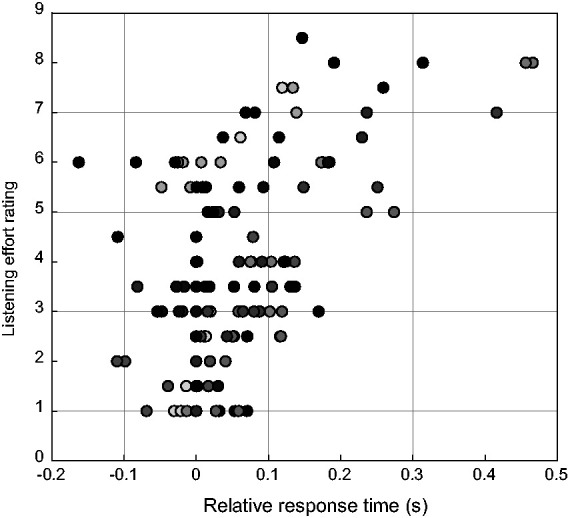
Scatterplot of the Mean Relative Response Times for Each Participant Per Condition Versus the Mean Listening Effort Rating for Each Participant Per Condition. Each marker represents the result of one condition per participant. Different participants are marked with a different gray tone.

A Pearson’s product moment correlation measurement of the estimated means for perceived listening effort and relative response time, per participant and per condition, resulted in a correlation coefficient of *R*_142_=0.54, *p* < .0001. There were no significant correlations between subjects within a condition. Figure 5 shows a scatterplot of the mean relative response times for each participant per condition versus the mean listening effort rating for each participant per condition. Each marker represents the result of one condition per participant. Different participants are marked with a different gray tone.

## Discussion

The main purpose of the current experiment was to test the hypothesis that NR is able to reduce response times in an auditory-only dual-task paradigm for HI listeners, the target group for using NR. We found a significant reduction in relative response times with increasing SNRs. Within the unprocessed conditions, response times were longer for speech in noise than for speech in quiet, although speech intelligibility was maximal for both conditions. This was previously shown for NH listeners by van den Tillaart-Haverkate et al. (2017). The current results show that the finding that response times are influenced by the amount of noise present also holds true for HI listeners. Statistical analysis showed a significant main effect of processing conditions, which is displayed in Figure 2B: At all SNRs, the relative response times for the processed conditions are shorter than for unprocessed signals. This suggests that there is a positive effect of NR on response times. However, the subsequent post hoc analysis revealed only a significant reduction of response time between IBM and the other two conditions at –5 dB SNR.

The decrease in response times with an increase in speech intelligibility is an effect that has been observed before (Baer et al., 1993; Gatehouse & Gordon, 1990), but we are especially interested in the effect of NR on response times at SNRs where speech intelligibility is maximal. In this so-called area of interest, the decreases in response times when applying NR were not significant. This finding is in contrast to previous results in NH listeners (van den Tillaart-Haverkate et al., 2017). A possible explanation for these contrasting results might lie in the marked reduction in relative response times for IBM at –5 dB SNR, which caused the interaction effect of NR with SNR. The SNRs that were used in this experiment were chosen to correspond with the SNRs used in van den Tillaart-Haverkate et al. (2017), to allow for direct comparison. However, NH listeners in their study had maximal intelligibility scores even at –5 dB SNR which is not the case in the HI group. Because we are interested in the effect of NR on response times in listening situations with maximal intelligibility, it may in retrospect have been unnecessary to include –5 dB SNR.

Furthermore, a direct comparison of the current results with those in the study by van den Tillaart-Haverkate et al. (2017) is complicated by another difference between the studies. As described in the Methods section, we chose to use the average of all processing conditions in quiet for determining relative response times, which deviates from the analysis used by van den Tillaart-Haverkate et al. (2017). Because all conditions in quiet are acoustically and subjectively indistinguishable, the variance in response time between these conditions should not be used to enlarge differences between conditions at other SNRs. We therefore feel that the current method of data analysis is more appropriate, albeit less suitable for discussing differences between NH and HI listeners.

When we compare the absolute response time data from HI listeners with that of NH listeners (see Figure 2A), the most prominent difference is that the group of HI listeners needs more time to respond than the group of NH listeners. We believe that this difference is mainly related to age effects. The mean age of the NH listener group in van den Tillaart-Haverkate et al. (2017) was 24 ± 4.15 years, on average 36 years younger than the HI listener group. It is well established that response times to tasks increase with age (Melzer & Oddsson, 2004; Verhaeghen & Cerella, 2002). Verhaeghen and Cerella (2002) report that reaction times of older adults can be described as a linear transformation of reaction times of younger adults. We thus assume that the impact of this age effect can be decreased considerably should this linear transformation be applied. Besides age, hearing loss might also play a role in the longer absolute response times for HI listeners. It is well known that sound perception is different for HI listeners (Huber et al., 2018), but little is known about how this can affect cognitive processes as measured in dual-task paradigms. Therefore, the effect of both hearing loss and aging needs to be considered in the test performance.

A further aim of this experiment was to study the relevance of the proposed measure in terms of validity and reliability and its contribution to the current understanding of listening effort. We evaluated validity using a self-report measure of perceived listening effort. This allows us to compare objectively measured effects with the subjective perception of listening effort. When comparing Figure 2B with Figure 3B, similar trends are visible for response times and perceived listening effort: An increase in SNR is accompanied by a decrease in relative response times or perceived listening effort. Also NR processing reduces both response times and the level of perceived listening effort. This observed trend is confirmed by the correlation coefficient (*R*_142_=0.54, *p* < .0001) between the perceived listening effort rating task and the arithmetic task. Alhanbali et al. (2019) analyzed multiple measures that are used for evaluating listening effort and found weak correlation coefficients between all of them. The authors did not include a response-time measure in their analysis. However, in comparison with their other reported correlations, our response time measure has a fairly high correlation with perceived listening effort. This high correlation supports the hypothesis that response times as such reflect at least one aspect of the concept of listening effort (Houben et al., 2013; van den Tillaart-Haverkate et al., 2017).

To analyze the test–retest reliability of the response time measurement, we assessed correlations in the response time measure between visits. For the group data, we found a strong correlation (*R*_10_=0.95, *p* < .0001) of the mean relative response time per condition between Visit 1 and Visit 2. This correlation suggests that the retest results did not systematically deviate from the test results. Figure 3A visualizes the positive correlation of the group results in a scatterplot. What is striking is that the data points are all but one above or on the *x* = *y* line. This implies that there is an effect of visit on relative response times. Indeed, the mixed effect model showed a small but significant effect of *visit* which means that a significant part of the total variance is explained by the test–retest measurement. Relative response times at the second visit were on average 0.014 s longer than at the first visit. This means that in the second visit, the differences between the quiet conditions and the conditions in noise were a little more pronounced. Note that we are not looking at a learning effect, because the relative data were determined within visits. Given the tiny effect size of the factor *visit* (*d* = 0.016), as interpreted according to Funder and Ozer (2019), and the high correlation between visits of the group-averaged relative response times, the response time measure was deemed to have a sufficient test–retest reliability.

The applicability of the arithmetic test at an individual level is currently not very promising. We found a moderate correlation (*R*_142_=0.42, *p* < .001) of relative response times per condition and per participant between Visit 1 and Visit 2. Individual results in Visit 1 were hardly reproducible in Visit 2, which is visualized in Figure 3B. If we were to correlate the absolute response times, this correlation would be strong. The absolute response times within participants were rather consistent, but this correlation does not give us information on the test–retest reliability of the relative response times. The weak correlation of the relative response times can be explained by the large within participant variability. From the mixed effects model, it was derived that for the arithmetic task 7.8% of the residual variance is explained by the between-participant variability. This means that a large proportion of the variances that are not explained by the fixed effects remain unexplained. This can be due to measurement errors or other undetermined factors such as within participant variability. On an individual level, the arithmetic task will have an even higher residual variance and is therefore inconclusive.

Another reason for the poor individual applicability of the arithmetic test could be the amount of response time data that is required for interpretable results. Brysbaert and Stevens (2018) recommends having at least 1,600 observations per condition for a properly powered response time experiment. In our experiment, there were only 120 observations per condition per individual which is not nearly enough for seeing an effect. When interpreting the group results, the data set is expanded to 1,440 observations per condition, which makes it possible to make an adequate analysis. In other words, the amount of individual data points should be largely expanded for interpreting the individual results. In practical terms, this would result in disproportionately long test time for participants. For now, we recommend to use the arithmetic task only for research on group effects rather than for individual effects, at least until the task is further optimized or more is known about the currently unexplained within-subject variance. For research on group effects, it should be noted that due to the nature of the test, the arithmetic task can only be used with speech materials consisting of digits.

The FUEL model states that cognitive-behavioral measures (such as the arithmetic task) are more closely related to the task-demand part of listening effort than to the motivational part. For the arithmetic task, task-demand is related to the listening situation (listening in noise) and the arithmetic calculation (summing two digits).The high accuracy of the results shows that the arithmetic sum is easy. Therefore, we assume that the difference in response time is determined more by the difficulty of the listening situation than the summing process itself. Although the arithmetic task is easy, it requires more mental resources than just repeating the digits and is therefore of added value in discriminating between the amount of mental effort that is required for the different listening situations (Houben et al., 2013; van den Tillaart-Haverkate et al., 2017).

The self-perceived listening effort rating and arithmetic task gave comparable results. This is not necessarily so for all setups or stimuli. Several studies have reported a low correlation between self-report and behavioral measures (Alhanbali et al., 2019; Dang et al., 2020; McGarrigle et al., 2014). An explanation for this low correlation could be because self-report measure can assess both motivational and task-demand aspects of listening effort (McGarrigle et al., 2014). It is plausible that in different applications (e.g., laboratory setup vs. real-life measurements, different background noises, research vs. clinical applications), the self-perceived listening effort rating task can switch from assessing the task-demand domain to assessing the motivation domain. This is less likely in the arithmetic task due to its objective nature. Both the objective and subjective perspective provide valuable information for evaluating listening effort, and therefore, they should not be used interchangeably.

Our results are inconsistent with findings from Sarampalis et al. (2009) who have reported a reduction in listening effort by applying an MMSE algorithm at an SNR where speech intelligibility was also affected (–6 dB SNR). Their study, however, lacks the inclusion of HI listeners, which could explain the differences in outcome. Desjardins and Doherty (2014), who did include HI listeners, also measured a reduction in listening effort in a more complex listening situation by using NR from commercial HAs. Both studies used response times in a visual dual-task paradigm in contrast to the auditory-only dual-task used in the current study. Given the differences in methods between the studies, regarding both participants and the type of NR processing, there is no reason to reject the hypothesis that using an auditory secondary task is also suitable for evaluating the effect of NR processing on listening effort.

We have established that the arithmetic task is adequate for evaluating the task-demand aspect of listening effort. The question remains why an expected effect of NR processing was not found. One possible explanation could be that there is a trade-off between noise attenuation and signal distortion. The MMSE algorithm that we used makes an imperfect estimation of the amount of noise and speech that is present in an acoustic signal. Therefore, it is inevitable that the algorithm will also distort the speech signal at the output. While the absolute reduction of noise level can facilitate a quicker response in the arithmetic task, the distortions might complicate the task causing longer response times. Brons et al. (2014b) found that HI listeners tolerate fewer audible distortions than NH listeners. This may explain why on average we found no effect of the MMSE algorithm. This trade-off is known to be a complicating factor in the interpretation of NR benefits, especially because of the large variability in individual preferences for NR settings (Neher, 2014; Reinten et al., 2019). Moreover, Neher (2014) concluded that executive functions can contribute to the interindividual variability in NR preferences. Other studies have also found a relationship between executive functions and susceptibility to distortions from HA signal processing (Arehart et al., 2013; Lunner, 2003). An executive function test to account for cognitive profiling could be a valuable addition to future experiments.

Another possible interpretation of the minimal effects of NR processing found in this study is related to our choice of background noise. Ng et al. (2015) used a behavioral task to measure the influence of NR on cognitive resources. The authors found that NR was able to improve recall of words spoken in a background of babble noise in the native language of the participants. This effect was not seen when a foreign language babble noise was used. These results imply that cognitive resources are affected more when informational masking is involved. Ohlenforst et al. (2018) tested the effect of NR on the measured peak pupil dilation in stationary noise and in four-talker babble noise, as a proxy for listening effort. They found a positive effect of NR on their proposed index of listening effort only for the four-talker babble noise. The positive effects of NR on peak pupil dilation when using stationary background noise were in line with the improvement in intelligibility of the signals, which is comparable to our results. In future experiments, one might consider measuring response times with different kinds of background noises.

## Conclusion

The response times measured during an auditory-only dual-task paradigm decrease significantly with increasing SNRs in HI listeners. In contrast to van den Tillaart-Haverkate (2017), we did not find a significant effect of the application of realistic NR to speech in noise on response times. The response times have a sufficient test–retest reliability and correlate with the subjective results of perceived listening effort rating. The arithmetic task therefore seems valid and reliable for using in evaluating the effect of the task-demand aspect of listening effort. In its current form, the arithmetic task is suitable for a research setting but less for clinical application due to the required amount of data and subsequent long test duration.
